# OsNPR3.3-dependent salicylic acid signaling is involved in recessive gene *xa5*-mediated immunity to rice bacterial blight

**DOI:** 10.1038/s41598-020-63059-8

**Published:** 2020-04-14

**Authors:** Guanghuai Jiang, Dedong Yin, Yue Shi, Zhuangzhi Zhou, Chunrong Li, Pengcheng Liu, Yanfeng Jia, Yanyan Wang, Zhenzhen Liu, Minxiang Yu, Xianghong Wu, Wenxue Zhai, Lihuang Zhu

**Affiliations:** 1 Center for Molecular Agrobiology,Institute of Genetics and Developmental Biology, Chinese Academy of Sciences, Beijing, 100101 China; 2 State Key Laboratory of Plant Genomics, Institute of Genetics and Developmental Biology, Chinese Academy of Sciences, Beijing, 100101 China

**Keywords:** Microbe, Biotic

## Abstract

Salicylic acid (SA) is a key natural component that mediates local and systemic resistance to pathogens in many dicotyledonous species. However, its function is controversial in disease resistance in rice plants. Here, we show that the SA signaling is involved in both pathogen-associated-molecular-patterns triggered immunity (PTI) and effector triggered immunity (ETI) to *Xanthomonas oryzae* pv. *Oryzae* (*Xoo*) mediated by the recessive gene *xa5*, in which OsNPR3.3 plays an important role through interacting with TGAL11. Rice plants containing homozygous *xa5* gene respond positively to exogenous SA, and their endogenous SA levels are also especially induced upon infection by the *Xoo* strain, PXO86. Depletion of endogenous SA can significantly attenuate plant resistance to PXO86, even to 86∆HrpXG (mutant PXO86 with a damaged type III secretion system). These results indicated that SA plays an important role in disease resistance in rice plants, which can be clouded by high levels of endogenous SA and the use of particular rice varieties.

## Introduction

Bacterial blight (BB) caused by *Xanthomonasoryzae pv. Oryzae* (*Xoo*) is a serious bacterial disease in rice-growing areas and can reduce the rice yield by up to 50% in some areas of Asia^1^. The most effective and economical management strategy for BB is the utilization of BB resistance genes in breeding. Of note, the rice-*Xoo* interaction has now become an important model system to study diseases in monocotyledonous plants^2^. Approximately 44 rice bacterial blight resistance genes (BBR genes) have been identified to date^3,4^. Of them, 10 genes, including *Xa21*, *Xa1*, *Xa27*, *xa5*, *xa13*, *Xa26*, *Xa10*, *Xa23*, *xa25* and *Xa4*, have been characterized and functionally analyzed^4,5^. The molecular mechanisms responsible for the resistance of these cloned genes is currently being investigated, and several signal components, including Xb3^6^, Xb15^7^, Xb24^8^, OsWRKY62^9^ and OsWRKY13^10^, COPT1, and COPT5^11^, have been identified in the signaling pathways mediated by the dominant resistant gene, *Xa21*, or recessive resistant gene, *xa13*. However, information is currently sparse concerning the resistance mechanisms of the recessive gene *xa5*. The *xa5* gene provides broad-spectrum resistance to strains of *Xoo* and encodes a small subunit of transcription factor IIA-like protein, which is structurally unlike the proteins encoded by other cloned disease resistance genes, especially recessive genes such as *mlo*^12^, *RRS1-R*^13^, *xa13*^14^ and *xa41*^3^. Although it can reduce resistance mediated by the *Xa27* gene^15^, the pyramid lines of the *xa5* gene and some other dominant genes such as *Xa21* and *Xa4* have a higher and wider spectrum resistance than the plants harboring only one BB resistance gene^1^. The dominant allele of *xa5* (*Xa5*) was shown to interact directly with the effectors from *Xoo* to activate the expression of susceptible genes and lead to rice disease^16^. Additionally, the resistance mediated by *xa5* was shown to result from the restricted bacterial movement^17^, which depended on the effectors of pathogens and correlated with the reduced expression of the cognate susceptible genes^18^. These contradictory reports indicate that the mechanisms of *xa5* and *Xa5* in bacterial blight resistance are complex.

In response to pathogen attacks, plants have evolved sophisticated defense mechanisms^19^. This system comprises three barriers. The first one is the physical protection, which includes hardening of the cell wall and the production of phytoalexins and antibiotic proteins^20,21^. When pathogens break through the first tier, the pathogen-associated molecular patterns (PAMPS) will be perceived by transmembrane pattern recognition receptors (PRRs), resulting in PAMP-triggered immunity (PTI)^19,22^, which composes a complex set of responses for a broad range of pathogens, including nonspecific and specific pathogens. To inhibit PTI, bacterial pathogens can deliver virulence effector proteins through the type III protein secretion system (T3SS) into plant cells^23^, and the third barrier is activated shortly thereafter. The reaction involves the recognition of effectors directly or indirectly by resistance proteins, is generally consistent with the “gene-for-gene” hypothesis and is termed effector-triggered immunity (ETI). ETI reactions often trigger the generation of reactive oxygen species and the rapid expression of pathogen-related (PR) genes and are accompanied by localized programmed cell death known as the hypersensitive response (HR) at the site of pathogen invasion. In fact, the three levels of immune responses are regulated by cross-communicating signal pathways involving Ca^2+^, ROS (reactive oxygen species) and NOS (reactive nitrogen species, namely, nitric oxide and its derivatives) bursts and phytohormones^24^. Among these signaling pathways, phytohormones play important roles in fine-tuning the responses, and salicylic acid (SA), jasmonic acid (JA) and ethylene (ET) are the three major defense hormones^25^.

JA and ET are generally required for defense against necrotrophs, whereas SA is involved in defense mechanisms against biotrophic pathogens^26^, especially in basal resistance or systemic acquired resistance (SAR)^27^. Increasing the endogenous levels or exogenous application of SA can induce the expression of pathogenesis-related (PR) genes in many dicotyledonous plants^28^. In contrast, transgenic plants expressing a bacterial salicylate hydroxylase (NahG) gene fail to accumulate SA following pathogen attack and cannot activate defense responses^29^ or the expression of PR genes^30^. Likewise, NahG can reduce the basal level of SA in rice, resulting in enhanced susceptibility against avirulent and virulent races of *M. grisea*^31^. PR genes are usually used as a marker to monitor the induction of SAR^28^, as its activation requires recruitment to its promoter of an SA-dependent transcriptional complex of TGA-NPR1^32^. The TGAs are basic-region leucine zipper (bZIP) transcription factors containing the TGACG-sequence-specific binding domain (TGA) and act as repressors or activators^32,33^. The Non Expressor of *PR* genes 1 (*NPR1*) is a transcriptional coactivator that was identified downstream SA in the defense response to pathogens^34^. NPR1 resides in the cytosol as oligomers under basal conditions and releases monomers into the nucleus after SA levels increase^35^. The localization of NPR1 in the nucleus is critical to activate *PR-1*^36^.

In the Arabidopsis genome, there are ten TGA-like proteins, seven of which are involved in defense against pathogens^37^. In addition, six NPR1-like proteins, including AtNPR1, have been characterized in Arabidopsis, among which AtNPR1, AtNPR3 and AtNPR4 act as receptors of SA to participate in pathogen defense^38–40^. In the rice genome, fifteen TGA-like and five NPR1-like proteins have been identified, among which rTGA2.1, OsNPR1 and OsNPR3 were shown to be involved in rice defense mechanisms^41–46^. There are at least two SA-signaling pathways in rice. In addition to the OsNPR1-dependent pathway, the WRKY45-dependent SA pathway has recently been identified too^47,48^. OsWRKY45 also plays important roles in rice bacterial blight and blast resistance^49^. Very interestingly, many rice cultivars have a much higher basal level of SA (8–37 μg/g fresh weight) than the elevated SA levels (500–2000 ng/g fresh weight) that can cause SAR in many infected dicotyledonous plants^31,50–52^. Although many studies have shown that rice shares most regulatory components in SA signaling pathways with dicots, most pathogen infections do not result in increases in the SA level in rice, and exogenously applied SA cannot induce *PR* gene expression and disease resistance in many rice cultivars^52^.

The two factors, the high levels of SA in rice and the use of particular rice varieties in related experiments, may cloud our understanding of the role of SA in rice disease resistance. Here, we describe for the first time the SA signaling pathway is involved in recessive gene *xa5*-mediated defense to BB, in which the paralog of OsNPR1, OsNPR3.3 plays an important role in a SA-dependent manner through interacting with TGAL11.

## Results

### The SA signaling may be involved in the *xa5*-mediated disease resistance to *Xoo*

**Figure 1:**
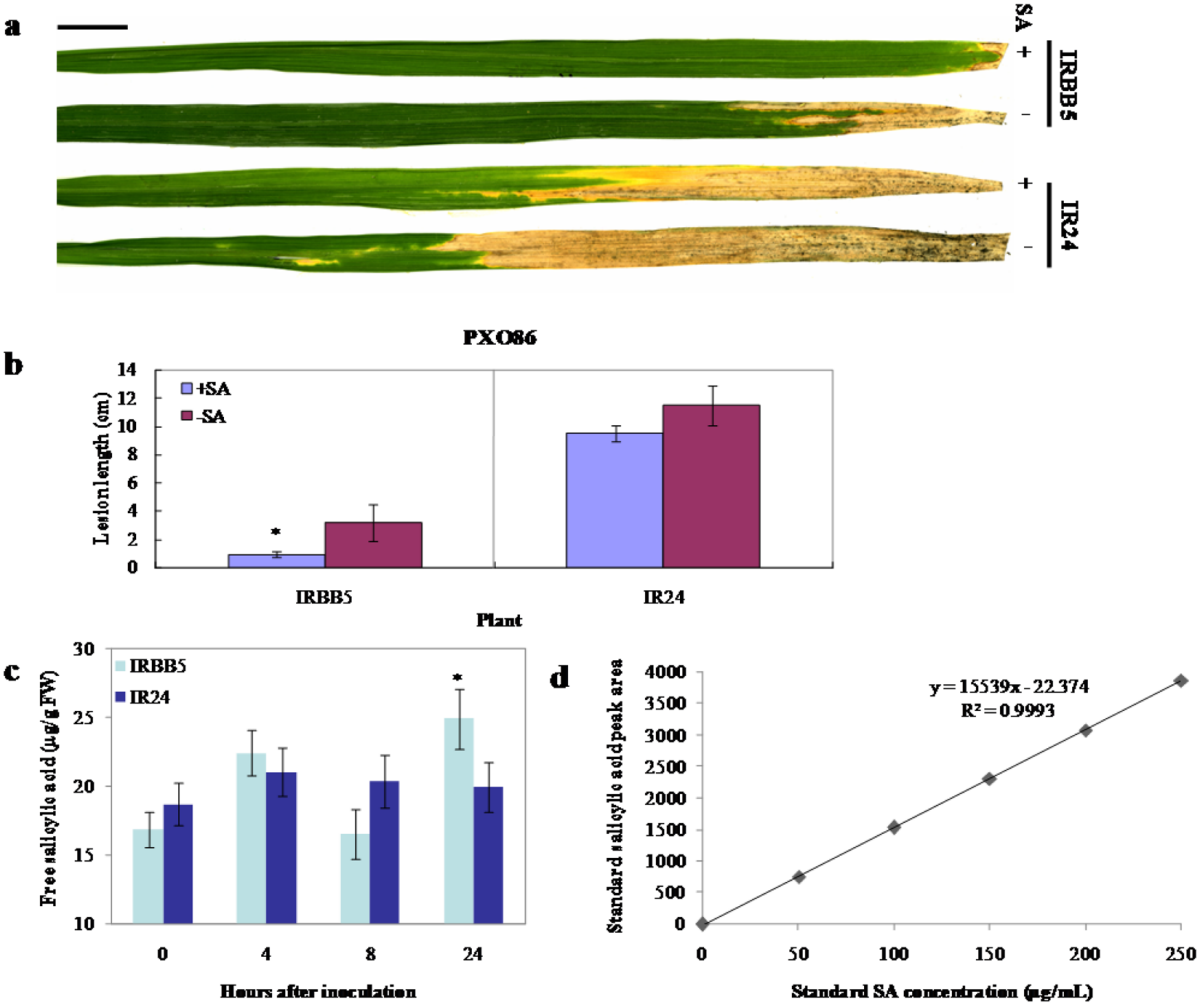
Influence of salicylic acid on bacterial blight resistance in rice plants to *Xoo* strain PXO86 and contents of SA in rice. (**a**) Resistance phenotype of rice plants at the tillering stage 14 dai. (**b**) Lesion length on leaves of rice plants at 14 dai. (**c**) Free SA levels in IRBB5 and IR24 inoculated with *Xoo* strain PXO86. (**d**) Standard curve of SA. The plants used in a and b were pretreated with 2 mM exogenous SA or H_2_O for one day, and then inoculated with PXO86. IRBB5 is the rice variety containing the homozygous recessive resistance gene *xa5*; IR24 is the susceptible near isogenic line of IRBB5.

Previous studies have shown that exogenous SA is unable to induce disease resistance in most rice plants^31,52^. However, we found that IRBB5, an *indica* variety containing the homozygous recessive resistance gene *xa5*, can respond strongly to SA treatment, while its susceptible near isogenic line, IR24^53^, cannot or shows a weak response (Fig. 1a,b). To study the action of SA on the disease resistance mediated by the *xa5* gene, we checked the free SA levels through reversed-phase high-performance liquid chromatography (RP-HPLC)^54,55^ in the leaves of IRBB5 and IR24 challenged with the differential *Xoo* strain PXO86. As shown in Fig. 1c, both IR24 (≈18.7 µg/g fresh weight) and IRBB5 (≈16.9 µg/g fresh weight) had higher basal SA levels, which were induced very quickly to their peaks of 21.08 and 22.5 µg/g fresh weight at 4 hours after inoculation (hai), and then declined to a lower level at 8 hai. Interestingly, the SA level in IRBB5 was induced again after 8 hours and reached a second peak of 25 µg/g fresh weight at 24 hai. The standard curve of the SA peak area shows a linear regression with R2 = 0.9993 (Fig. 1d). These results indicated that the SA signaling might be involved in *xa5*-triggered disease resistance to *Xoo*.

### Expression of salicylate hydroxylase in IRBB5 attenuates plant resistance to *Xoo*

**Figure 2:**
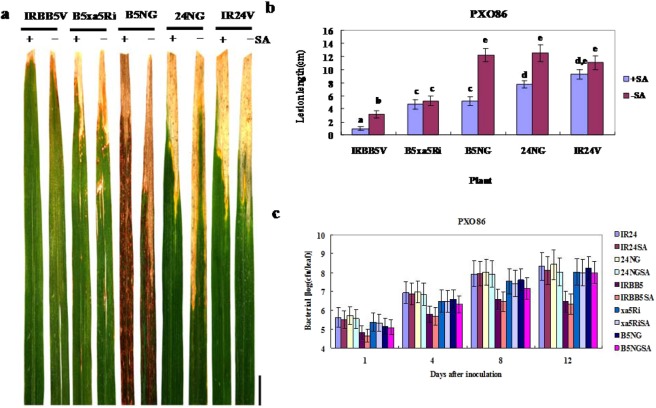
Salicylic acid is involved in the disease resistance of IRBB5 to *Xoo* strain PXO86. (**a**) Resistance of IRBB5, IR24, B5xa5Ri, 24NG and B5NG plants at the flourishing tillering stage. NahG-dependent lesion formation is shown in the leaves of B5NG plants. The phenotype survey is carried out at 14 dai. The scale bar is 2 cm. (**b**) Lesion lengths on the leaves of rice plants at 14 dai. Values are the mean ± SD of three independent biological replicates. Letters denote significant differences based on analyses of plants treated with SA (+SA) and H_2_O (−SA) using the Tukey significance difference test (p < 0.05). (**c**) Bacterial populations of PXO86 in rice plants. Number of bacteria in 1-cm leaf sections from the cut sites were measured at 1, 4, 8 and 12 dai. Bars are means ± standard error. These plants were pretreated with 2 mM SA or H_2_O for one day and then inoculated with PXO86. B5NG and 24NG are the T_1_ transgenic plants overexpressing the bacterial *nahG* gene in IRBB5 and IR24 respectively; B5xa5Ri is the xa5 RNAi plant in IRBB5 background; IRBB5V and IR24V are the transgenic plants with pCAMBIA1300 vector in IRBB5 and IR24 respectively.

To further determine the role of SA in the disease resistance mediated by *xa5*, we generated three kinds of transgenic plants. Two SA-deficient rice plants, B5NG and 24NG, were produced by overexpressing the bacterial *nahG* gene in IRBB5 and IR24, respectively. The other was the RNA interfering plant (xa5RNAi) in the IRBB5 background, in which loss or reduction of *xa5* expression was achieved using double-stranded RNA. Consistent with previous studies^31,56^, SA levels were significantly reduced to 150–500 ng/g fresh weight and 300–810 ng/g fresh weight in B5NG and 24NG plants, respectively. Interestingly, *nahG*-dependent brownish lesions gradually appeared from the base of the leaves after the four-leaf stage and expanded to the whole leaf in B5NG plants, while those in 24NG mainly appeared in old leaves at very limited amounts (Fig. 2a). In addition, the B5NG plants grew more slowly than 24NG and the control plants. At the four-leaf and adult stage, the heights of B5NG plants were approximately 12 cm and 50 cm, respectively, which was only half of the height of the wild type plants and 24NG. At the flourishing tillering stage, these transgenic plants were pretreated with 2 mM SA and then inoculated with *Xoo* strain PXO86 after 24 hours. Exogenous SA application improved the resistance of these plants to various degrees; however, the resistance of B5NG and xa5RNAi plants was largely compromised compared with the wild type IRBB5 at 14 days after inoculation (dai) (Fig. 2a,b). Correspondingly, the populations of PXO86 in the leaves of B5NG and xa5RNAi almost reached the levels in plants with IR24 background from the 4th dai (Fig. 2c). Moreover, the formation of brownish lesions became more severe on the leaves of B5NG plants. These findings indicated that the SA signaling was involved in the disease resistance mediated by the *xa5* gene.

### SA signaling is involved in PTI

**Figure 3:**
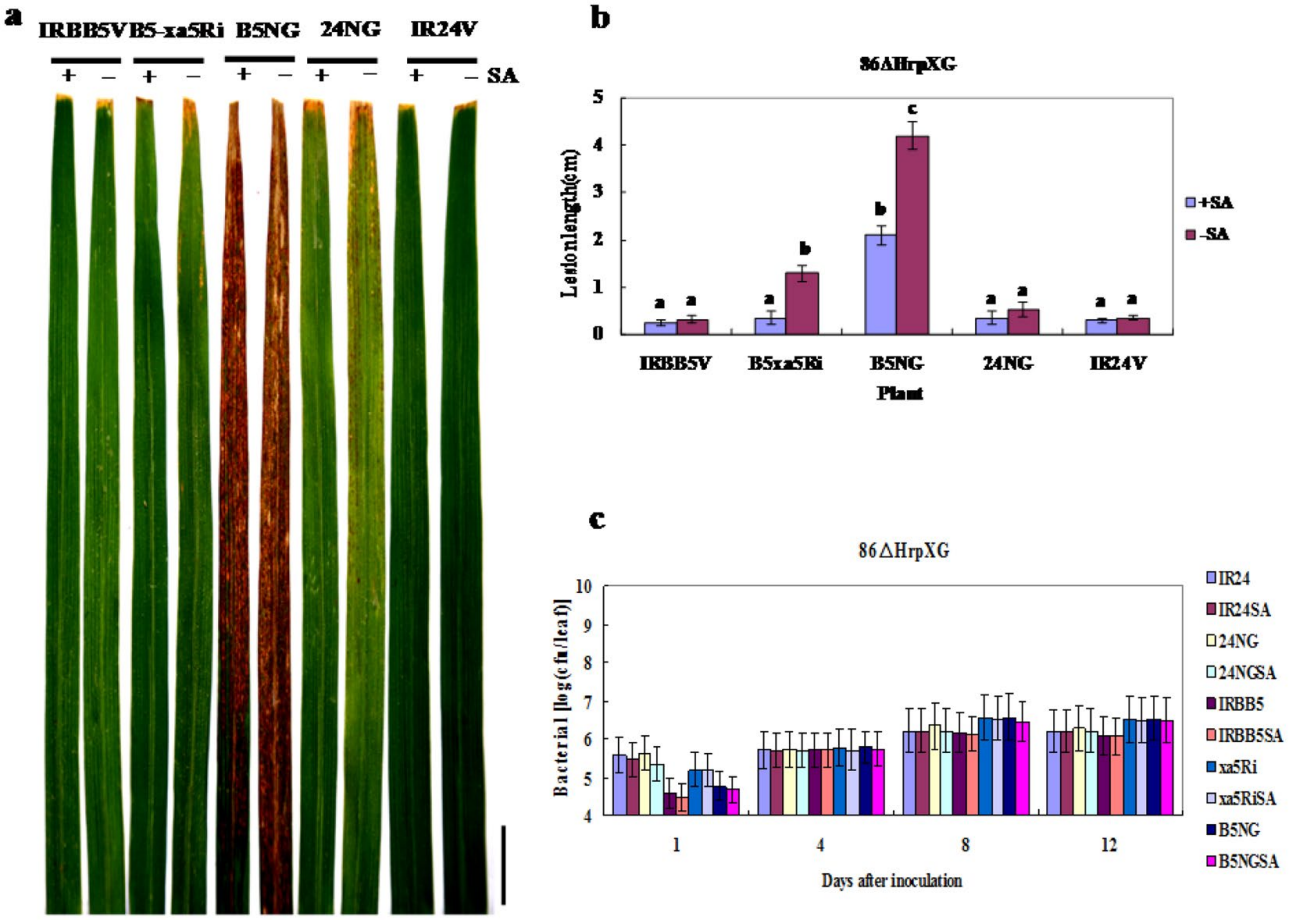
Salicylic acid is involved in xa5-mediated PTI. (**a**) Resistance of IRBB5, IR24, 24NG, B5xa5Ri and B5NG plants to *Xoo* T3SS mutant strain 86∆HrpXG. (**b**) Lesion length on leaves of rice plants at 14 dai treated with 86∆HrpXG. Values are the mean ± SD of three independent biological replicates. Letters denote significant differences based on analyses of plants treated with SA (+SA) and H_2_O (−SA) using the Tukey significance difference test (p < 0.05). (**c**) Bacterial populations of 86∆HrpXG in rice plants. Number of bacteria in 1-cm leaf sections from the cut sites were measured at 1, 4, 8 and 12 dai. Bars are means ± standard error. These plants were pretreated with 2 mM SA or H_2_O at the flourishing tillering stage for one day, and then inoculated with 86∆HrpXG. B5NG and 24NG are the T_1_ transgenic plants overexpressing the bacterial *nahG* gene in IRBB5 and IR24 respectively; B5xa5Ri is the *xa5* RNAi plant in IRBB5 background; IRBB5V and IR24V are the transgenic plants with pCAMBIA1300 vector in IRBB5 and IR24 respectively. The scale bar is 3 cm.

In *Xanthomonas* bacteria, T3SS is critical for their full virulence and bacterial colonization in host plants. The T3SS proteins are encoded by hypersensitive response and pathogenicity (hrp) genes, the expression of which is controlled by the two key regulators HrpX and HrpG^57^. To ascertain whether SA signaling is involved in PTI mediated by the *xa5* gene, we inoculated IR24, IRBB5 and their *nahG* transgenic plants with the T3SS mutant strain 86∆HrpXG, which was derived from PXO86 and the DNA fragment including the tandem *HrpX* and *HrpG* gene (the accession number of the PXO86 complete genome sequence in GenBank is CP007166.1, http://www.ncbi.nlm.nih.gov/nuccore/CP007166.1) was replaced with a kanamycin-resistance gene through marker exchange (Supplementary Fig. S1a-c). The resistance phenotype of leaves and multiplication of 86∆HrpXG in these plants were analyzed after inoculation. As shown in Fig. 3a–c, almost no symptoms were observed on the leaves of these plants, except B5NG and xa5RNAi that were not treated with exogenous SA, indicating that SA signaling is also involved in PTI, and *xa5* may not be related to the response.

### *OsNPR1, OsNPR2* and *OsNPR3* are responsive to *Xoo* in rice

**Figure 4:**
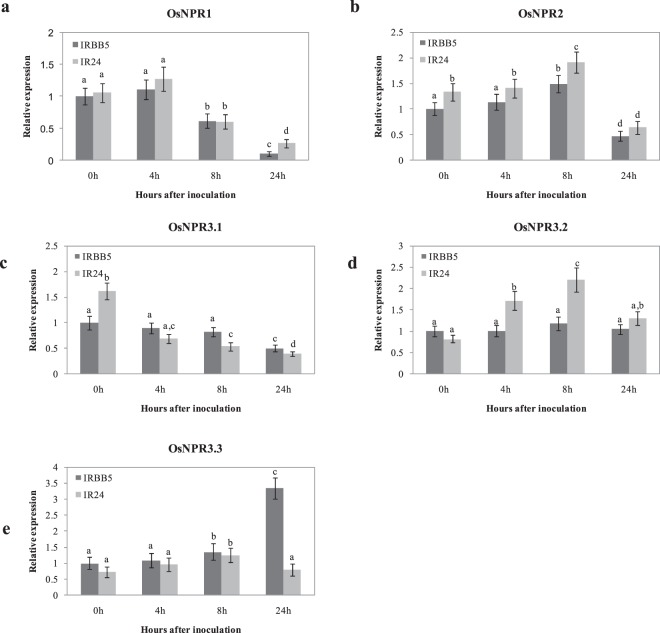
Expression analysis of NPR1 like genes in rice inoculated with *Xoo* strain PXO86. (**a**) LOC_Os01g09800.1, (**b**) LOC_Os01g56200.1, (**c**) LOC_Os03g46440.1, (**d**) LOC_Os03g46440.2, and (**e**) LOC_Os03g46440.3 in IRBB5 and IR24. Transcript levels were determined by quantitative real-time RT-PCR. Values were normalized to the expression of each gene in IRBB5 that was not treated with *Xoo*. The rice actin gene served as the internal control. Bars represent the average ± SD of three biological replicates. Different letters above columns indicate significant differences at P < 0.05 as determined by a one-way ANOVA followed by post hoc Tukey honest significant difference (HSD) analysis.

Considering the high SA levels in both IRBB5 and IR24, as well as the involvement of SA in *xa5*-mediated PTI and ETI in response to *Xoo*, we examined the expression patterns of *OsNPR1*-like genes in IRBB5 and IR24 inoculated with the *Xoo* strain PXO86. Five *NPR1* like genes were described previously in rice, but their nomenclature was somewhat confusing. For example, *OsNPR4* and *OsNPR5* were respectively designated with different locus numbers in previous reports^41,46^. In fact, there are seven *NPR1*-like gene loci in the rice genome according to the recently released TIGR database: *LOC_Os01g09800*, *LOC_Os01g56200*, *LOC_Os03g46440*, *LOC_Os01g61990*, *LOC_Os01g72020*, *LOC_Os11g04600* and *LOC_Os12g04410*. Among these loci, *LOC_Os01g56200*, *LOC_Os03g46440*, *LOC_Os01g61990* and *LOC_Os01g72020* have 2 or 3 alternative splicing isoforms, and *LOC_Os11g04600* encodes the same protein as *LOC_Os12g04410*. Comparison of these proteins showed that LOC_Os01g56200.2, LOC_Os01g61990.1, LOC_Os01g61990.2, LOC_Os01g72020.1, LOC_Os01g72020.2 and LOC_Os11g04600.1, without the typical ankyrin repeat domain or NPR1/NIM1-like defense protein C terminus (NPR1_like_C), should not truly be considered as NPR1-like proteins (Supplementary Fig. S2a). Thus, there are five typical NPR1-like proteins, in a strict sense, in rice, namely OsNPR1, OsNPR2, OsNPR3.1, OsNPR3.2 and OsNPR3.3 (Supplementary Table S1). The phylogenetic tree analysis showed that the 11 proteins respectively encoded by the seven rice NPR1-like gene loci and six Arabidopsis NPR1-like proteins could form three groups, and the OsNPR1-3 proteins had a relatively close relationship with the AtNPR1-4 proteins, indicating that the OsNPR1-3 proteins might be involved in disease resistance (Supplementary Fig. S2b). Figure 4a–e shows that *OsNPR1-3* genes all respond to the *Xoo* strain PXO86. Their expression levels were suppressed (*OsNPR3.1*) or induced initially and then declined at 8 hai (*OsNPR1*, *OsNPR2*, *OsNPR3.1* and *OsNPR3.2*) in both IR24 and IRBB5, whereas the expression of *OsNPR3.3* was specifically induced in IRBB5 after 24 hai. These results suggest that among the *OsNPR* genes, *OsNPR3.3* may play a critical role in the resistance signaling pathway mediated by the *xa5* gene.

### OsNPR3.3 plays an important role in bacterial blight resistance mediated by the *xa5* gene

**Figure 5:**
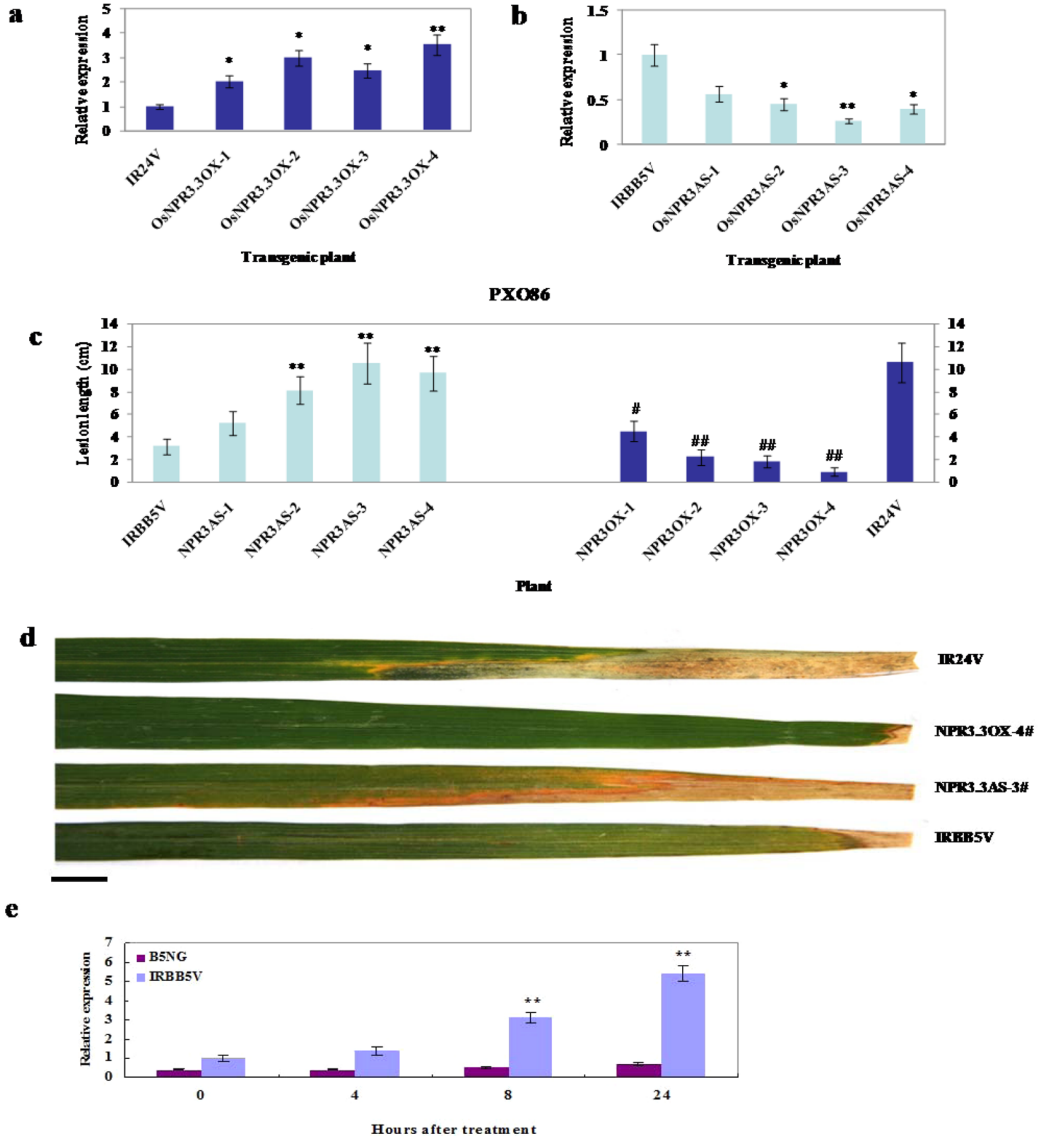
Effect of OsNPR3.3 in bacterial blight resistance mediated by the *xa5* gene. (**a**,**b**) expression analysis of *OsNPR3.3* in *OsNPR3.3*-overexpressing (OsNPR3.3OX) (**a**) and antisense-suppressing (OsNPR3.3AS) (**b**) T_1_ transgenic plants, respectively. (**c**) Lesion lengths of OsNPR3.3OX and OsNPR3.3AS T_1_ transgenic plants inoculated with PXO86. The leaves were photographed at 14 dai. (**d**) Phenotypes of *NPR3.3* transgenic plants at the flourishing tillering stage. NPR3.3OX-4# and NPR3.3AS-3# are *OsNPR3.3* overexpressing and antisense-suppressing T_1_ transgenic plants, respectively. (**e**) Expression analysis of *OsNPR3.3* in IRBB5 and B5NG T_1_ transgenic plants inoculated with *Xoo* strain PXO86. Transcript levels were determined by quantitative real-time RT-PCR. Values were normalized to the expression of *OsNPR3.3* in IRBB5V or IR24V, respectively. The rice *actin* gene served as the internal control. Bars represent the average ± SD of three biological repeats. Asterisks or pound signs indicate a statistically significant difference compared with untreated IRBB5 (*P < 0.05 and **P < 0.01). B5NG is the T_1_ transgenic plant overexpressing the bacterial *nahG* gene in IRBB5. IRBB5V and IR24V are the transgenic plants with pCAMBIA1300 vector in IRBB5 and IR24 respectively. The scale bar is 2 cm.

To elucidate the putative function of OsNPR3.3 in the *xa5*-mediated defense response to *Xoo*, we generated two binary plasmids with overexpression (OsNPR3.3OX) and antisense suppression (OsNPR3.3AS) of its cDNA under the control of the constitutive CaMV 35S promoter. These plasmids were introduced into IR24 and IRBB5, respectively, by Agrobacterium-mediated transformation. No obvious morphological changes were observed in these transgenic plants compared with the control plants. The expression levels of *OsNPR3.3* in these transgenic plants were examined by qRT-PCR. Sixteen independent lines carrying the OsNPR3.3OX plasmid showed increased expression of *OsNPR3.3* (at least two-fold) compared with the transformed IR24 containing the pCAMBIA1300 vector (IR24V) (Fig. 5a). Additionally, 9 suppression lines showed approximately 90% to 60% expression reduction of *OsNPR3.3* compared with the transformed IRBB5, containing the pCAMBIA1300 vector (IRBB5V) (Fig. 5b). These transgenic plants were then challenged with the *Xoo* strain PXO86 at the tillering stage. The OsNPR3.3OX transgenic plants exhibited shorter lesions compared with the control plant IR24V, whereas the OsNPR3.3AS plants showed longer lesions compared with the control plant IRBB5V (Fig. 5c,d). Moreover, we examined the *OsNPR1* and *OsNPR2* in these OsNPR3AS lines, but their expression patterns were the same as those in the control plant IRBB5 (Supplementary Fig. S3). To further investigate whether SA was required for activation of *OsNPR3.3* in the disease resistance mediated by *xa5*, we analyzed the *OsNPR3.3* expression patterns in the SA-deficient transgenic plant B5NG inoculated with the *Xoo* strain PXO86. As shown in Fig. 5e, *OsNPR3.3* expression relied strongly on SA accumulation. The expression of *OsNPR3.3* was induced very quickly in IRBB5 after 4 hours of treatment with SA, while that in B5NG plants was not significantly induced. In addition, the resistance of OsNPR3.3AS plants was slightly attenuated in response to 86∆HrpXG (Supplementary Fig. S4). These data suggest that *OsNPR3.3* participated in the disease resistance mediated by the *xa5* gene in an SA-dependent manner.

### OsNPR3.3 is a nuclear targeted protein with weak transcriptional activation

**Figure 6:**
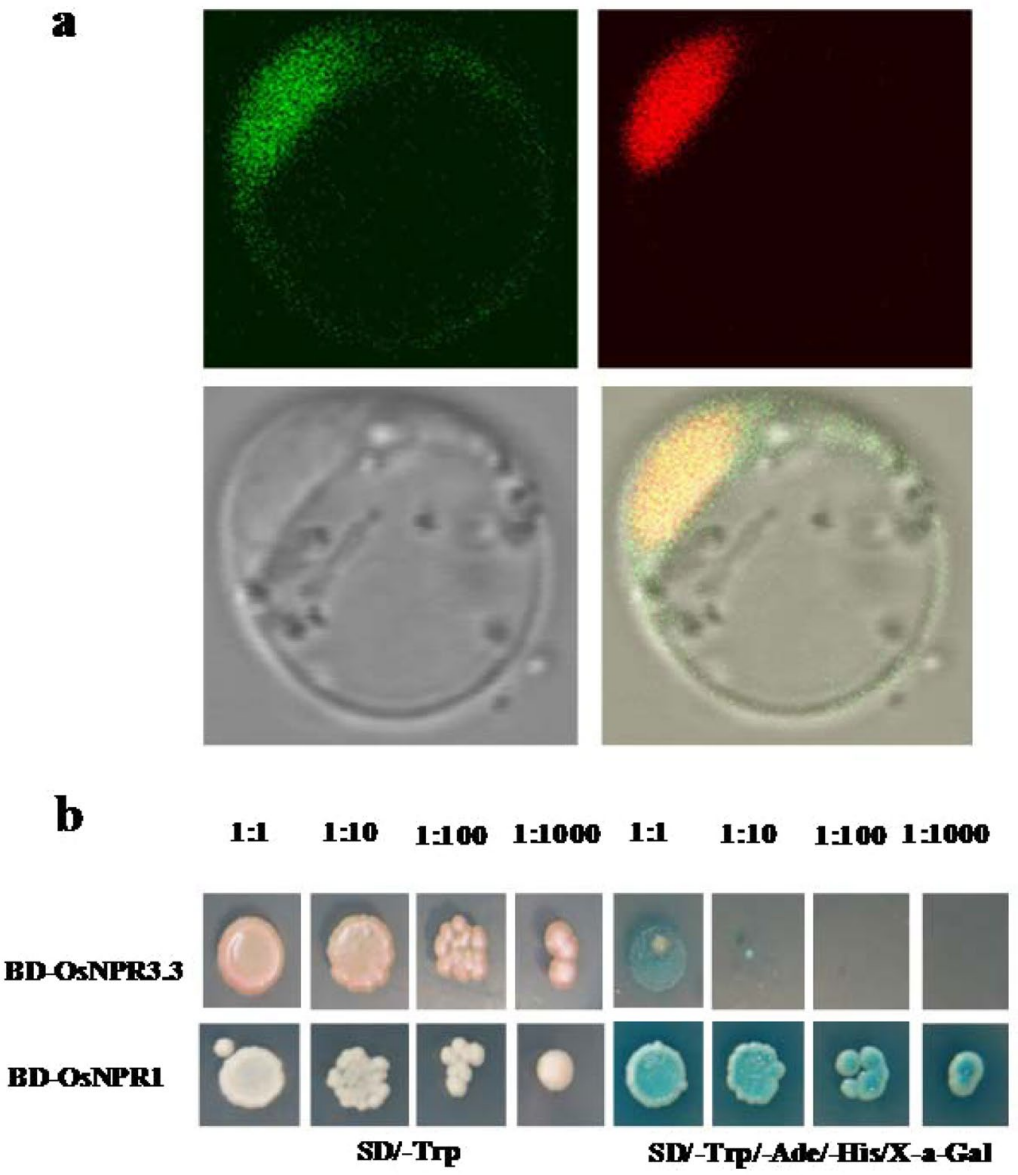
Subcellular localization and transcriptional activation analysis of OsNPR3.3. (**a**) Nuclear localization of OsNPR3.3. Protoplasts were transfected with the OsNPR3.3-YFP construct. OsABF1-RFP was used as a nuclear localization marker. The YFP (left) and RFP (right) signals in the top panel are green and red, respectively. The bright field (left) and merged (right) images are shown at the bottom. The yellow signal shows that the YFP and RFP overlapped very well in the nucleus. (**b**) Transcriptional activation test of OsNPR3.3 in yeast cells. OsNPR1 was used as a positive control. All the yeast cells were 10-fold serially diluted and spread on culture medium. BD is the DNA binding domain of the yeast GAL4 transcription factor.

The OsNPR3.3 protein is predicated to be a nuclei targeted or nucleocytoplasmic protein by WoLF PSORT (http://www.genscript.com/wolf-psort.html). To determine its subcellular localization, the full-length coding sequence (CDS) was fused in frame to the N-terminus of yellow fluorescent protein (35S-OsNPR3.3-YFP) and co-transformed with a nuclear marker, OsABF1 tagged with a red fluorescent protein (OsABF1-RFP)^58^, into IRBB5 protoplasts. The yellow fluorescent signal merged very well with the red signal of OsABF1-RFP (Fig. 6a), indicating that it was localized to the nucleus. Likewise, OsNPR3.1 and OsNPR3.2 were also shown targeted to the nucleus (data not shown), indicating that the difference in the carboxyl terminus of OsNPR3 proteins did not affect their localization.

To determine whether OsNPR3.3 had transcriptional activation as OsNPR1, its entire CDS was then fused in frame to the C-terminus of the DNA binding domain of GAL4 to create a bait in the plasmid of pGBK-T7(BD-OsNPR3.3), which was transferred into the yeast strain YH109 containing four reporter genes, His3, ADE2, lacZ and MEL1 (Clontech). OsNPR1 was used as the positive control (BD-OsNPR1). The data presented in Fig. 6b show that the cells harboring BD-OsNPR1 could grow on the two selected media, SD/-Trp and SD/-Trp/-Ade/-His/X-α-gal. Although the cells containing BD-OsNPR3.3 can grow well on the selected medium, SD/-Trp, they grew slowly and infirmly on the SD/-Trp/-Ade/-His/X-α-gal medium, suggesting that OsNPR3.3 activated transcription of the reporter genes at low levels.

### The OsNPR3.3 cooperates with TGAL11 to participate in bacterial blight resistance mediated by the *xa5* gene

**Figure 7:**
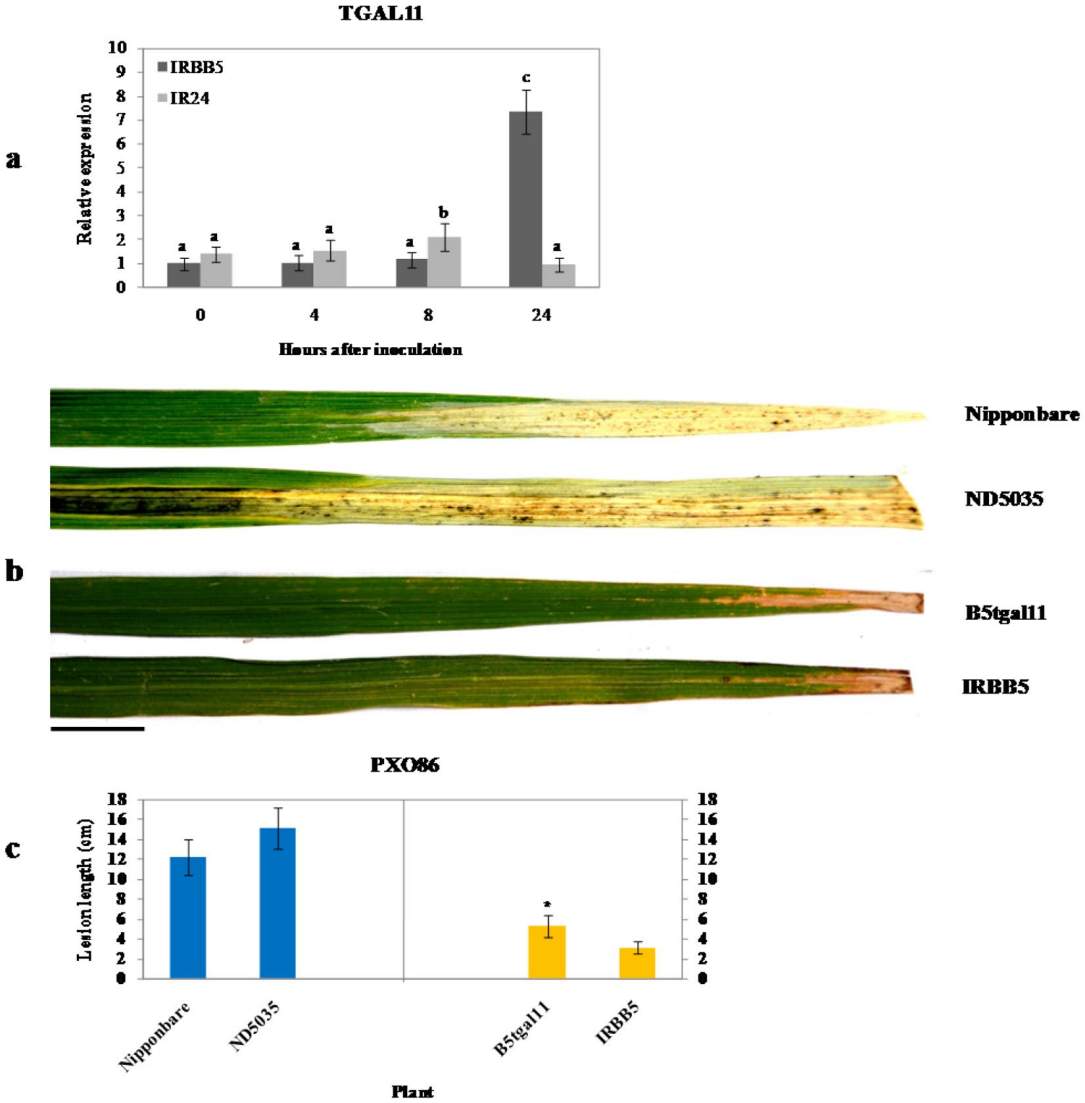
Effect of TGAL11 in bacterial blight resistance mediated by the *xa5* gene. (**a**) Expression analysis of *TGAl11* in IRBB5 and IR24 inoculated with *Xoo* strain PXO86. Transcript levels were determined by quantitative real-time RT-PCR. Values were normalized to the expression of each gene in IRBB5 that was not treated with *Xoo*. The rice actin gene served as the internal control. Different letters above columns indicate significant differences at P < 0.05 as determined by a one-way ANOVA followed by post hoc Tukey honest significant difference (HSD) analysis. (**b**) Phenotypes of TGAL11 mutant plants inoculated with PXO86 at the flourishing tillering stage. The scale bar is 3 cm. (**c**) Lesion lengths of TGAL11 mutant plants inoculated with PXO86. ND5035 and B5tgal11 are TOS17 insertion mutant plants in Nipponbare and IRBB5 background respectively with destroyed *TGAL11* gene. The leaves were photographed at 14 dai. Bars represent the average ± SD of three biological replicates.

OsNPR3 (NH3) has been reported to interact with eight rice TGA proteins (rTGA2.1, rTGA2.2, rTGA2.3, rLG2, TGAL1, TGAL2, TGAL4 and TGAL11) in both yeast and rice cells^42^. To ascertain whether the eight TGA genes were copartners of OsNPR3.3 participating in the bacterial blight resistance mediated by the *xa5* gene, we examined their expression in IRBB5 and IR24 challenged with the *Xoo* strain PXO86 at the tillering stage by qRT-PCR analysis. As shown in Fig. 7a and Supplementary Fig. S5, the expression of *TGAL1* was initially suppressed and then upregulated at 8 hai in both IRBB5 and IR24; *TGAL4* was downregulated in both IRBB5 and IR24; *TGAL2*, *rTGA2.2* and rTGA2.3 were upregulated in both IRBB5 and IR24; *rTGA2.1* was slightly induced at 4 hai and declined substantially at 8 hai in IRBB5; and *rLG2* showed no marked change in IRBB5. Only *TGAL11* declined initially and then increased significantly at 8 hai in IRBB5, in keeping with the expression pattern of *OsNPR3.3* (Fig. 4e). In addition, the phylogenetic relationship analysis showed that TGAL11 had significant similarity to TGAL4, TGAL5, rLG2 and AtTGA9 (Supplementary Fig. S6a). AtTGA9 is involved in ROS-mediated responses to the bacterial PAMP flg22 in Arabidopsis^27^. These findings imply that TGAL11 may cooperate with OsNPR3.3 to participate in *xa5*-mediated bacterial blight resistance. To verify this speculation, a Nipponbare-derived Tos17 insertion mutant line ND5035 was used as a pollen recipient in a cross with IRBB5 to obtain the mutant line carrying the homozygous destructive *TGAL11* in the IRBB5 background (B5tgal11). The ND5035 mutant line had a Tos17 insertion in the third intron, which resulted in breakage of the *TGAL11* gene (Supplementary Fig. S6b,c). A total of 20 plants derived from B5tgal11 were inoculated with the *Xoo* strain PXO86 at the tillering stage. The lesion lengths of leaves in these B5tgal11 plants were significantly increased compared with IRBB5 at 14 dai (Fig. 7b,c). These plants were also inoculated with 86∆HrpXG, but they displayed no symptoms (Supplementary Fig. S6d). Altogether, these findings indicated that TGAL11 was involved in OsNPR3.3-mediated disease resistance.

### SA signaling in *xa5*-*OsNPR3.3*-mediated bacterial blight resistance is independent of *OsNPR1* and *OsWRKY45*

**Figure 8:**
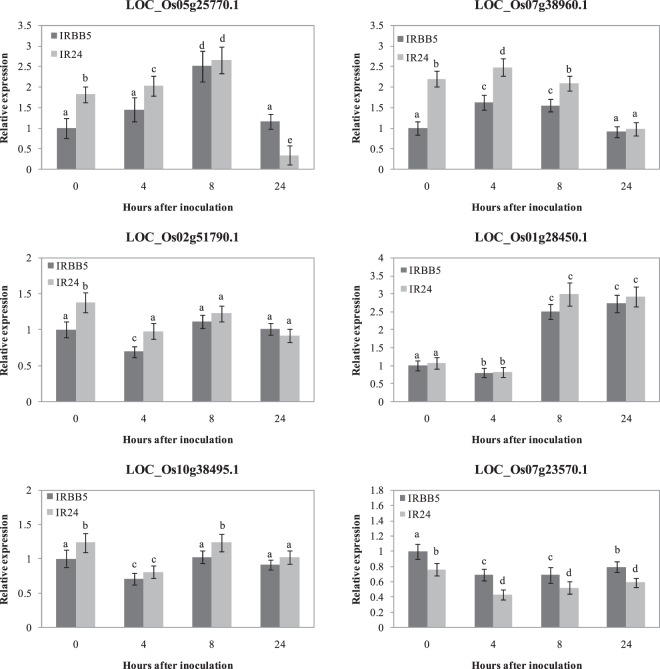
Expression analysis of *OsWRKY45* and SA-responsive genes, including *OsNPR1*-dependent and *OsWRKY45*-dependent, in IRBB5 and IR24. Transcript levels were determined by quantitative real-time RT-PCR. Values were normalized to the expression of each gene in IRBB5 that was not treated with *Xoo*. The rice *actin* gene was used as the internal control. Bars represent the average ± SD of three biological replicates. Different letters above columns indicate significant differences at P < 0.05 as determined by a one-way ANOVA followed by post hoc Tukey honest significant difference (HSD) analysis.

Previous studies have shown that the SA signaling in rice branches into OsNPR1- and OsWRKY45-dependent pathways^48^. In addition to the *OsNPR1* gene (Fig. 4a), we also analyzed the expression patterns of *OsWRKY45* (*LOC_Os05g25770.1*) and SA-responsive genes including *OsNPR1*-dependent (*LOC_Os07g38960.1*, *LOC_Os02g51790.1* and *LOC_Os01g28450.1*) and *OsWRKY45*-dependent (*LOC_Os10g38495.1* and *LOC_Os07g23570.1*)^45,47^ in IRBB5 and IR24. *OsWRKY45* showed a similar expression pattern between the two plants; namely, it was induced at 4 hai and peaked at 8 hai, followed by a decline at 24 hai (Fig. 8). Interestingly, the expression patterns of these *OsNPR1*- or *OsWRKY45*-dependent genes all showed no significant differences between the two plants (Fig. 8). Moreover, we produced transgenic plants in the susceptible Nipponbare background overexpressing *OsNPR1* (OsNPR1OX), *OsNPR3.1* (OsNPR3.1OX) and *OsWRKY45* (OsWRKY45OX). They were subjected to *Xoo* strain PXO86 to test the functions of *OsNPR1*, *OsNPR3.1*, *OsNPR3.2* and *OsWRKY45* in disease resistance. As shown in Supplementary Fig. S7, all OsNPR1OX, OsNPR3.1OX and OsNPR3.2OX plants showed significant resistance to PXO86, while the OsWRKY45OX plants did not. These findings indicate that the salicylic acid signaling is involved in the rice bacterial blight resistance mediated by the *xa5* gene, which is dependent mainly on *OsNPR3.3*.

## Discussion

Previous studies have shown that many plant species, such as Arabidopsis, tobacco and cucumber, contain low basal levels of salicylic acid, which acts as a key signaling molecule in the activation of local or systemic acquired resistance, while rice plants contain high levels of endogenous SA, and SA levels cannot be increased by bacterial or fungal pathogens, suggesting that SA may not play a signaling role in rice disease resistance^52^ but functions as an antioxidant molecule to protect rice from oxidative stress through modulating the redox balance^31^. However, in this study, we showed that although the rice varieties IRBB5 and IR24 both contained high levels of SA, the levels could be increased by *Xoo* strain PXO86 at the early stage of infection, followed by a decline after 4 hai. Moreover, IRBB5 had a second much stronger response to the infection after 8 hai, indicating that the early small increase in SA was a result of the response to PAMPs and the second significant induction was the result of response to effector.

In addition to its function as an antioxidant^59^ in the modulation of the cell redox balance^31^ or as scavenger of hydroxyl radicals to protect plants against catalase inactivation by H_2_O_2_^60^, our results suggested that SA is also a signaling molecule in the regulation of both PTI and ETI in rice. Although the key molecule, *OsNPR1*, in the signal transduction pathway from SA to defense responses was suppressed in both IRBB5 and IR24, one of its paralogs, *OsNPR3.3*, was dramatically induced in IRBB5 by *Xoo* strain PXO86. Overexpression of *OsNPR3.3* in the susceptible plant IR24 enhanced its resistance to PXO86. Considering that the application of exogenous SA to IRBB5 could further shorten the lesion length, we reasoned that *OsNPR3.3* was activated to substitute for *OsNPR1* and played the central role in the SA signaling pathway in IRBB5. Simultaneously, *OsTGAL11* was induced by *xa5* to participate in disease resistance to *Xoo* through interactions with OsNPR3.3. In fact, overexpression of *OsNPR1* and *OsNPR3.1*/*OsNH3* can confer rice disease resistance to bacterial blight or blast^41,45,46^. Therefore, we speculated that the universally inability of SA to activate local defense or SAR in rice is due to the suppression of *OsNPR1* in most rice varieties without major resistance gene(s) under the pressure of competition and evolution between rice and pathogens. The appearance of the recessive gene *xa5* activated the SA signaling pathway to lead to disease resistance or SAR in rice by upregulating the expression of *OsNPR3.3*.

Because *OsNPR1* and *OsNPR3.1* were usually expressed at lower levels and the SA signaling was blocked, endogenous SA continues to increase and maintains a high level in most rice varieties. The high level of endogenous SA maintains rice with a high reducing status. Depletion of endogenous SA in *nahG* transgenic rice plants overexpressing bacterial salicylate hydroxylase upsets the redox balance and elevates the levels of superoxide and H_2_O_2_, finally resulting in the lesion mimic phenotype and growth retardation. Interestingly, most lesion mimic plants have been reported to possess disease resistance to pathogens, and their lesion formation is associated with an overaccumulation of reactive oxygen species, such as superoxide radical and H_2_O_2_^61^. On the other hand, the *nahG* transgenic plants with lesion mimic exhibit increased susceptibility to pathogens, despite their levels of superoxide and H_2_O_2_^31,56,62^. Consistent with these previous reports, the transgenic B5NG plants over-expressing *nahG* exhibited resistance attenuation to *Xoo*. However, the basic resistance attenuation and lesion development in B5NG plants was different from that of 24NG plants, in which the lesions mainly appeared in old leaves at very limited amounts. The various expression of *NahG* led to the 24NG plant having higher basal SA levels than B5NG, which might accounts for the phenotypic difference. When SA levels are high in rice plants, SA is essential to modulate cell redox balance^31^, it can acquire superoxide dismutase activity through interacting with iron^63^ and function as an antioxidant^59^, or protect catalase from inactivation and reduced the accumulation of H_2_O_2_^60^_._ On the contrary, SA functions mainly as signaling molecule to activate the basic resistance in lower levels. In addition, H_2_O_2_ is a Janus-faced molecule. At high concentrations, it can cause hypersensitivity reactions or cell dehydration by loosening the cell wall; at low concentrations, it can strengthen the cell wall or act as a signaling molecule^64^.

Taken together, the present studies show that the SA signaling pathway is involved in *xa5*-mediated bacterial blight resistance by upregulation of *OsNPR3.3* and *TGAL11*. Once IRBB5 plants suffer infection by an incompatible *Xoo* strain, SA synthesis and disease resistance mediated by *xa5* and *OsNPR3.3*, are quickly activated, finally resulting in the disease resistance response, which may partially account for the broad-spectrum resistance conferred by the *xa5* gene.

## Materials and methods

### Plant materials and growth conditions

The rice (*Oryza sativa*) *indica* cultivar IR24 and its near isogenic line IRBB5 containing homozygous *xa5* gene; *japonica* cultivar Nipponbare were used in this study. IRBB5 is resistance to the Philippine *Xoo* strain PXO86^53^. All rice plants were grown in rice fields in Beijing (40°2′N, 116°2′E). Rice leaves were sprayed with 2 mM SA or diluted water as control 24 hours before inoculation with *Xoo* strain.

### Measurement of salicylic acid

Free salicylic acid was extracted from 0.5 g 4-week-old rice seedlings inoculated with *Xoo* strain PXO86, and quantified by reverse phase high performance liquid chromatography (RP-HPLC) as described previously^65,66^. Briefly, the tissue was homogenized in 90% methanol. After centrifugation, the pellet was reextracted with 100% methanol. The combined supernatants were repeated frozen and centrifuged. The supernatant was added 2 mL of 5% trichloroacetic acid and extracted for three times with 3 mL of ethylacetate-cyclopentane (1:1 in volume). The combined organic phase was dried in a speed vacuum. The residue was resuspended in 1.5 mL of 70% methanol and passed through Diamonsil C_18_ column (5 µm, 250 mm × 4.6 mm, Dikma Technologies) pretreated with 70% methanol. The free SA was then separated from conjugated SA through organic extraction with 2 volumes of ethylacetate-cyclopentane (1:1) for three times. The combined organic phase containing the free SA was then dried by vacuum dryer. The dried extract was suspended in 1.5 mL of 70% methanol, and passed through 70% methanol-pretreated C_18_ column. The elution fraction was dried by vacuum dryer and resuspended in 250 µl initial solution. The solution containing free SA was filter-sterilized and then analyzed by HPLC on Agilent HP1100 (Agilent company, http://www.agilent.com).

### Protein structure and phylogenetic analysis

All the protein sequences were obtained Rice Genome Annotation Project (http://rice.plantbiology.msu.edu/). Their structures were on-line analyzed through National Center for Biotechnology Information (NCBI) (https://www.ncbi.nlm.nih.gov). The sequence alignment and molecular phylogeny were analyzed by using DNAstar software (version 5.00, www. dnastar.com).

### Construction of T3SS mutant through Marker-exchange

The *HrpX* and *HrpG* are closely connected with each other in the *Xoo* genome. The construction of deletion mutant of HrpX-HrpG was performed as described previously^67^. Firstly, three DNA fragments were PCR amplified by using primers listed in Supplementary Table S2. The 805 bp *HrpX* and 828 bp *HrpG* genome DNA fragments were from *Xoo* strain, PXO86, the other is for 982 bp *aphA1* gene from pUC-4K plasmid^68^. Secondly, the three DNA fragments were mixed as templates to amplify the overlapping DNA fragment with *aphA1* gene. PCR products were gel purified and cloned into pGEM-Teasy (Promega) with NotI and MluI to produce the plasmid, pGK-HrpXG. Then the plasmid was introduced into PXO86 and selected on PSA medium with Kanamycin.

### Functional complementation of *HrpX*-*HrpG* mutant

A 3064 bp genomic DNA fragment containing the full length *HrpX* and *HrpG* genes was PCR amplified by using primer pair, HrpXcF/ HrpGcR. The PCR product was cloned into pGEM-Teasy and sequenced. Then the confirmed 3064 bp fragment was cut with HindIII and XhoI into HindIII-SalI digested pHM1 vector to yield the functional complementation plasmid. The T3SS mutant strain of PXO86 was transferred with the plasmid and selected on the spectinomycin plate. The positive clones were picked to test their virulence on rice plants.

### *Xoo* inoculation

The rice plants at tillering stage were inoculated with *Xoo* strain, PXO86 by using the leaf clip method^53^. Each sample was treated for 5 plants with 10^5^ cells/ml as the initial concentration.

### Quantitative real time RT-PCR (qRT-PCR) analysis

Total RNAs were extracted from rice leaves using Trizol (Invitrogen). After treating with RNA-free DNase I (Promega), the RNAs were reverse-transcribed to cDNAs using M-MLV (Promega) with oligo(dT)_15_. qRT-PCR was performed using SYR Premix EX Taq (TaKaRa) on a BIO-RAD CFX96 system. The rice *Actin* gene (X16280) was used as the internal control. Each gene was analyzed using three biological replicates. Significance was analyzed using a *t*-test. The sequences of these primers are presented in Supplementary Table S2.

### Plasmid construction and rice transformation

The complete coding regions of *OsNPR1*, *OsNPR3.1*, *OsNPR3.2* and *OsNPR3.3* were obtained from IRBB5 through RT-PCR, and inserted into pCAMBIA1300 (http://www.cambia.org) driven by a 1669 bp 35S promoter from pEZR(K)-LN^69^ to produce the overexpression plasmids (p1300S-OsNPR3.1, p1300S-OsNPR3.2, p1300S-OsNPR3.3 and p1300S-OsNPR1); The complete coding region of *OsNPR3.3* were cloned into pZh01^64^ vector for antisense suppression plasmids, pZh01-OsNPR3.3. For the subcellular localization of OsNPR3.3, the full-length coding sequence (CDS) of OsNPR3.3 without stop codon was cloned into the pSAT6-EYFP-N1 vector^70^. The *xa5* RNA interference vector was constructed using a 390-bp cDNA fragment from *Xa5* gene (accession no. AK065182) as described previously^69^.The sequences of the primers used here are provided in Supplementary Table S2.

### Yeast experiment

Yeast two-hybrid assays were performed in accordance with the Matchmaker Gold Yeast Two-Hybrid System (http://www.clontech.com). The complete coding regions of *OsNPR3.3* and TGA like genes were cloned into the pGBKT7. In the same way, the complete coding region of *OsNPR3.3* was cloned into pGADT7 vector for its transcription activation analysis. OsNPR1 in pGBKT7 was used as the positive control. The interactions of murine p53 with SV40 large T-antigen was used as the positive control for the interaction between proteins. The interaction between human lamin C and an unrelated protein T was used as a negative control. Transformation efficiency was estimated by plating an aliquot of transformation mixtures onto SD/-Leu-Trp medium. The interactions between proteins were confirmed on SD/–Ade/–His/–Leu/–Trp supplemented with 125 ng/ml Aureobasidin A (AbA) and 40ug/ml X-α-gal (Invitrogen).

### Reporting summary

Further information on research design is available in the Nature Research Reporting Summary linked to this article.

## Supplementary information

Supplementary Information.

## Data Availability

The authors declare that all data supporting the findings of this study are available within the paper and its supplementary information. Hao, Z. Title. Figshare https://doi.org/10.6084/m9.figshare.853801 (2014).
