# Supplementary material for: OsNPR3.3-dependent salicylic acid signaling is involved in recessive gene xa5-mediated immunity to rice bacterial blight
Source: Sci Rep. 2020 Apr 14;10:6313. doi: 10.1038/s41598-020-63059-8 (PMC7156675; doi:10.1038/s41598-020-63059-8)
Supplement: Supplementary file 1 — Supplementary Information. [file 41598_2020_63059_MOESM1_ESM.docx]

**OsNPR3.3-dependent salicylic acid signaling is involved in recessive gene *xa5*-mediated immunity to rice bacterial blight**

Guanghuai Jiang^1,3^**,** Dedong Yin^2,3^ **,** Yue Shi^1^ **,** Zhuangzhi Zhou^2^ **,** Chunrong Li^1^ **,** Pengcheng Liu^1^**,** Yanfeng Jia^1^ **,** Yanyan Wang^1^ **,** Zhenzhen Liu^1^ **,** Minxiang Yu^2^ **,** Xianghong Wu^2^ **,** Wenxue Zhai^1^ & Lihuang Zhu^2^

^1^Center for Molecular Agrobiology,Institute of Genetics and Developmental Biology, Chinese Academy of Sciences, Beijing 100101, China

^2^State Key Laboratory of Plant Genomics, Institute of Genetics and Developmental Biology, Chinese Academy of Sciences, Beijing 100101, China.

^3^These authors contributed equally: Guanghuai Jiang, Dedong Yin

**Correspondence and requests for materials should be addressed to W.Z. (email:** [**wxzhai@genetics.ac.cn**](mailto:wxzhai@genetics.ac.cn)**) or L.Z. (email:** [**lhzhu@genetics.ac.cn**](mailto:lhzhu@genetics.ac.cn)**)**

**Supplementary Information**

**Supplementary Figure S1**

**Supplementary Figure S2**

**Supplementary Figure 3**

**Supplementary Figure 4**

**Supplementary Figure 5**

**Supplementary Figure 6**

**Supplementary Figure 7**

**Supplementary Table S1.** NPR1-like proteins in rice genome

(http://rice.plantbiology.msu.edu/)

| **Name used before** | **NPR1-like gene locus** | **alternative splicing isoforms** | **Name used in this paper** | **ankyrin repeat domain** | **NPR1_like_C**  **domain** |
| --- | --- | --- | --- | --- | --- |
| **OsNPR1/NH1** | ***LOC_Os01g09800*** | **LOC_Os01g09800.1** | **OsNPR1** | **yes** | **yes** |
| **OsNPR2/NH2** | ***LOC_Os01g56200*** | **LOC_Os01g56200.1** | **OsNPR2** | **yes** | **yes** |
|  |  | LOC_Os01g56200.2 | no | no | no |
| **OsNPR3/NH3** | ***LOC_Os03g46440*** | **LOC_Os03g46440.1** | **OsNPR3.1** | **yes** | **yes** |
|  |  | **LOC_Os03g46440.2** | **OsNPR3.2** | **yes** | **yes** |
|  |  | **LOC_Os03g46440.3** | **OsNPR3.3** | **yes** | **yes** |
| OsNPR4 | *LOC_Os01g61990* | LOC_Os01g61990.1 | no | yes | no |
|  |  | LOC_Os01g61990.2 | no | yes | no |
| OsNPR5/NH4 | *LOC_Os01g72020* | LOC_Os01g72020.1 | no | yes | no |
|  |  | LOC_Os01g72020.2 | no | yes | no |
| NH5 | *LOC_Os11g04600* | LOC_Os11g04600.1 | no | yes | no |
| NH5 | *LOC_Os12g04410* | LOC_Os12g04410.1 | no | yes | no |

**Supplementary Table S2.** Primers used in this paper

| **Primer** | **Forward (5’to 3’)** | **Reverse (5’ to 3’)** | **Length of PCR production** | **Restriction enzyme** |
| --- | --- | --- | --- | --- |
| qActin | gagctacgagcttcctgatgg | aatgccagggaacatagtgg | 214bp | No |
| OsNPR1 | caagctgaaagaagggaccc | cagaacccaggttaaattcc | 193bp | No |
| OsNPR2 | gatggccagagtgcaatgag | ggtgacagaatcttccactg | 162bp | No |
| OsNPR3.1 (N3UF/N3.1R) | tgaaagtggcacagcagagg | gtgatagcttccctttcttg | 188bp | No |
| OsNPR3.2 (N3UF/N3.2R) | tgaaagtggcacagcagagg | gtgcgtcgcgtcgaccatg | 239bp | No |
| OsNPR3.3 (N3UF/N3.3R) | tgaaagtggcacagcagagg | gcgaggtcgactcatcttgg | 171bp | No |
| OsWRKY45 | aggaggaggtgctgagcag | agacaacctcgtcgttcttg | 174bp | No |
| Xa5Ri | actagtggtaccgattccaaactactcagcc | gagctctaccattaacataggatcc | 390BP | SpeI-KpnI/BamHI-SacI |
| OsNPR1 | gaattcgaggcctcctcctcgcctc | ggatccggagccaagaaatcatctc | 2113bp | EcoRI/BamHI |
| OsNPR3.1 | tcccacttcgctgctccc | cttggataaatagttggaattcc | 2466bp | BamHI/EcoRI |
| OsNPR3.2 | gaattcatggagacgtccaccataag | ggatcccgcgcgacgcgatctccccctccg | 1752bp | EcoRI/BamHI |
| OsNPR3.3 | ctcccacttcgctgctccc | cttaaaccgaacagtacacc | 3618bp | BamHI/EcoRI |
| OsNPR3.3_CDS | ggaattcatggagacgtccaccataag | ggatccggacttggtgatgaagacgcag | 1722bp | EcoRI/BamHI |
| OsNPR1_CDS | agaattctctagaatggagccgccgaccagcc | aggatccggtacctctccttggtcgaatggcc | 1746bp | EcoRI-XbaI/BamHI-KpnI |
| rTGA2.1 | gtaaactaggaacgctcgag | aagccacagcgaactcaaag | 164bp | No |
| rTGA2.2 | gtcaaatggctatggccatg | aagccagagagaactcaggg | 185bp | No |
| rTGA2.3 | gtgcgcttcttgtgataag | gaatcaagtcctttgcagg | 178bp | No |
| rLG2 | agcaggctgacaatctgagg | ttgcagctcggttcctgtgg | 197bp | No |
| TGAL1 | aagctcaagggtgtggcag | gccagcaagctgctgctctg | 168bp | No |
| TGAL2 | aagctcaagggtgtggcag | ggtcagcattttgcctccgg | 189bp | No |
| TGAL4 | tttgaggcagcagactatcc | ctgcaatgcttagctctcc | 182bp | No |
| TGAL11 | gaaggctttgtcatacagg | tgccaccaatatctctcgg | 174bp | No |
| rTGA2.1_CDS | cccgggtatggcagatgctagttcaagg | ggatccttactcccgtggcctagcaag | 1005bp | SmaI/BamHI |
| rTGA2.2_CDS | cccgggtatggcagatgctagttcgagg | ggatccttactcccgtggcctagcaag | 1002bp | SmaI/BamHI |
| rTGA2.3_CDS | gaattcatggcagatatgagccctagg | ggatccctattctttcggccgagcaag | 990bp | EcoRI/BamHI |
| rLG2_CDS | catatgatggtgcaaggtgaggagag | cccgggaaatcctgagtactgattctg | 1617bp | NdeI/SmaI |
| TGAL1_CDS | gaattcatggagggtggtaggctagg | ggatccttattcccttggacgggcgag | 1419bp | EcoRI/BamHI |
| TGAL2_CDS | gaattcatggagggtggtaggctagg | ggatccggtcagcattttgcctccgg | 1092bp | EcoRI/BamHI |
| TGAL4_CDS | gaattcatgggagaagctagcagtag | cccgggtcagaaggctgaatattgg | 1455bp | EcoRI/SmaI |
| TGAL11_CDS | catatgaagcttatgggagaggctaggagagg | ggatcctcagaaagctgaaaattgg | 1470bp | NdeI-HindIII/BamHI |
| HrpX(HrpXF/HrpXR) | gcggccgcctgcagtgcgcgcccggtc | actgacccactttcatctgggcc | 805bp | NotI/MluI |
| aphA1(KF/KR) | ggcccagatgaaagtgggtcagtgtgtctcaaaatctctgatg | ctttgtcttgcagcgcgagctctacaaccaattaaccaattc | 982bp | No |
| HrpG(HrpGF/HrpGR) | gagctcgcgctgcaagacaaag | actagttggctgatcggtgcag | 828bp | SacI/SpeI |
| HrpXHrpG(HrpXcF/ HrpGcR) | gaagcttcaacaaaccgccacggcagg | tctcgagagatcgcacacctgcagcgg | 3064bp | HindIII/XhoI |
